# Amiodarone Hepatotoxicity with Absent Phospholipidosis and Steatosis: A Case Report and Review of Amiodarone Toxicity in Various Organs

**DOI:** 10.1155/2013/201095

**Published:** 2013-05-20

**Authors:** Adela Cimic, Joseph Sirintrapun

**Affiliations:** Department of Pathology, Wake Forest Baptist Health, Medical Center Boulevard, Winston-Salem, NC 27157, USA

## Abstract

We present the first description of amiodarone toxicity in the liver without phospholipidosis or steatosis. In doing so, we will review the various effects of amiodarone toxicity in various organs. The patient is a young adult who had cardiac reconstruction as a child for transposition of the great vessels. A needle biopsy was taken due to elevated liver enzymes. Her ALT was 188 U/L (5–50) and AST 162 U/L (5–50). Alkaline phosphatase, total bilirubin, protein, and albumin were within normal limits. A serologic panel for viral hepatitis was negative. Antinuclear antibodies were positive at 260; however, anti-smooth muscle antibody and anti-mitochondrial antibody were negative. A protein electrophoresis showed a slightly elevated beta globulin 2 level of 0.5. Quantitative immunoglobulin levels were within normal limits except for a slightly elevated IgA 409 mg/dL (60–350). Liver ultrasound was unremarkable. The clinical differential was broad and included hepatic congestion along with autoimmune hepatitis. Sections showed only ballooned hepatocytes with Mallory-Denk bodies and perisinusoidal fibrosis. Arrival to the diagnosis was possible only after careful review of the patient's medications. After discontinuation of amiodarone, the patient's liver enzymes returned to normal levels.

## 1. Introduction

Determining possible etiologies in drug-induced liver injury (DILI) can be challenging. Very often, the diagnosis is made based on the clinical scenario and histopathology. Even when caused by the same agent, manifestations of DILI can vary significantly. For instance, some drugs (e.g., statins, minocycline, nitrofurantoin, and infliximab) cause idiosyncratic hepatocellular or cholestatic liver injury with some patients and autoimmune hepatitis in others [1].

Amiodarone is reported to cause various subtle clinical and morphologic effects in various organs. Because of the wide spectrum of subtle clinical and morphologic effects caused by amiodarone, it is often challenging even to arrive at the possibility of amiodarone-induced tissue injury.

Amiodarone is a potent antiarrhythmic agent (iodinated benzofuran derivative) which causes elevated liver enzymes in up to 30% of patients and steatohepatitis in 1-2% of patients [2–4]. The majority of cases display liver enzyme abnormalities within 24 h of intravenous infusion. Even low oral dosing (200 mg daily) may trigger steatohepatitis with cumulative use [5]. 

Amiodarone-induced hepatotoxicity is characterized by steatosis, enlarged hepatocytes, inflammation, fibrosis, and lamellar lysosomal inclusion bodies representing phospholipidosis [6]. Two potential explanations for the accumulation of tissue phospholipids include reduced phospholipid degradation because of direct action of amiodarone to inhibit phospholipases (especially lysosomal phospholipase) or binding of amiodarone to phospholipids rendering the drug-phospholipid complex more resistant to phospholipases [7–10]. Occasionally, jaundice is the major clinical presentation, showing morphologically hepatocellular necrosis and fibrosis. Such cases are noted to have poor prognosis [11]. 

We present the first case of drug (amiodarone-) induced liver injury without phospholipidosis or steatosis. In doing so, we will also review the literature on the morphology of amiodarone toxicity in various other organs in addition to the liver.

## 2. Report of a Case

A 33-year-old African American female with D-transposition of the great vessels underwent a Mustard procedure as a child. She had since developed recurrent bouts of atypical atrial flutter with failure of multiple antiarrhythmic regimens including cardioversion. She denied alcohol use. Her medications included lisinopril, metoprolol, Coumadin, vitamins, and amiodarone (400 mg daily). Her amiodarone intake was understated because she would intermittently take it for extended periods of time and then go off when there was resolution of the arrhythmia. Past medical history included hypothyroidism, anemia, and history of a pilocytic astrocytoma with subsequent stroke. Interestingly, her hypothyroidism was clinically suspected to be due to amiodarone toxicity.

The patient was referred to a gastroenterologist with new onset of liver enzyme elevation one month prior to biopsy. Her ALT was 188 U/L (5–50) and AST 162 U/L (5–50). Alkaline phosphatase, total bilirubin, protein, and albumin were within normal limits. Antinuclear antibodies were elevated at 260; however, anti-smooth muscle antibody and anti-mitochondrial antibody were negative. A protein electrophoresis showed a slightly elevated beta globulin 2 level of 0.5. Quantitative immunoglobulin levels were within normal limits except for a slightly elevated IgA 409 mg/dL (60–350). At that juncture, the clinical differential was broad and included passive hepatic congestion along with autoimmune hepatitis. The liver ultrasound was unremarkable, and a liver biopsy was performed.

**Figure 1:**
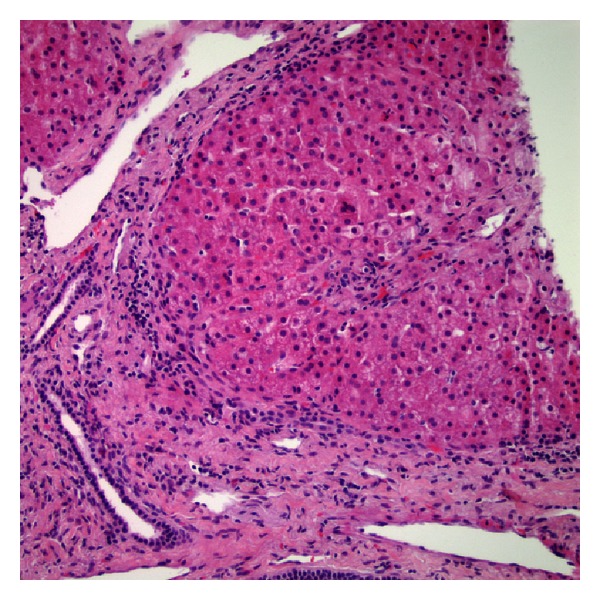
Minimally inflamed portal tracts, with subtle ductular proliferation.

**Figure 2:**
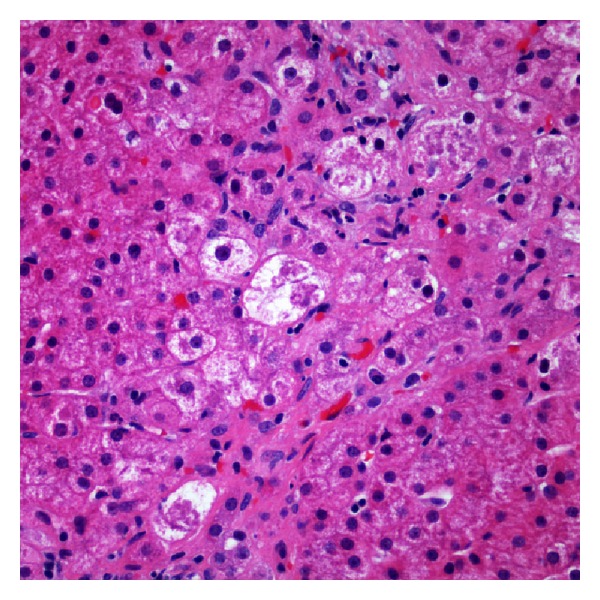
Marked ballooned hepatocytes with Mallory-Denk bodies without steatosis.

**Figure 3:**
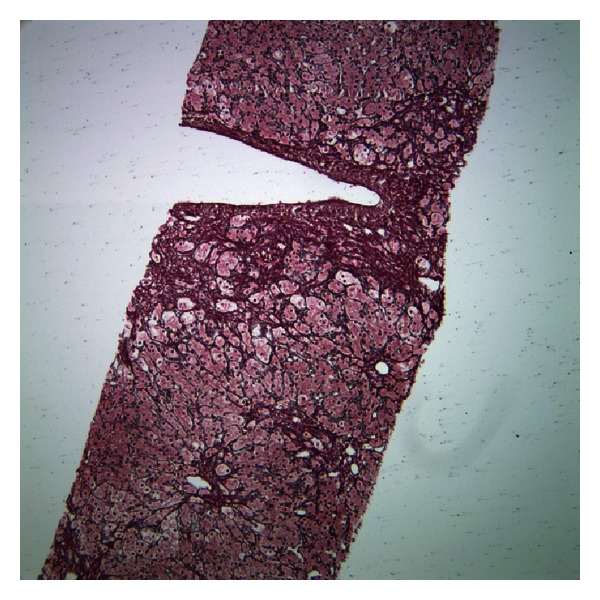
Extensive remodeling with zone 3 to 3 bridging (reticulin).

**Figure 4:**
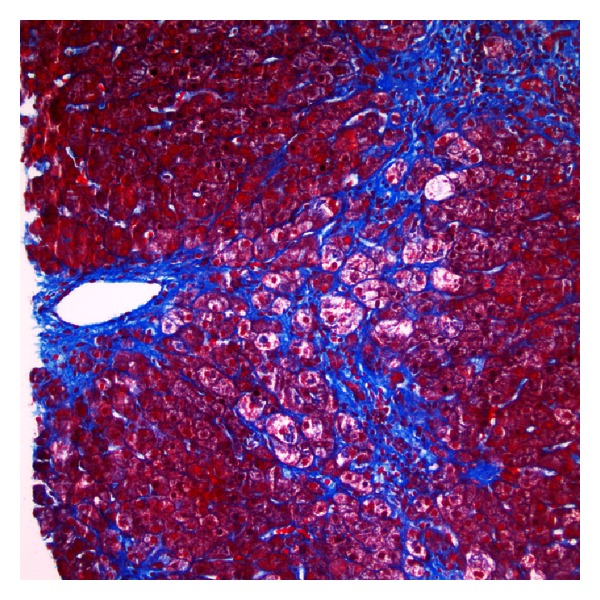
Prominent perisinusoidal fibrosis and ballooned hepatocytes with Mallory-Denk bodies (trichrome).

Histologic examination of the biopsy showed the portal tracts with minimal inflammation and a subtle ductular reaction (Figure 1). There was presence of ballooned hepatocytes and Mallory-Denk bodies (Figure 2). Reticulin and trichrome staining showed extensive remodeling with extensive perisinusoidal fibrosis and zone 3 to 3 bridging (Figures 3 and 4). Curiously, no phospholipidosis or steatosis was observed. There were no globules noted on Periodic Acid-Schiff Diastase (PASD) and the iron stains were negative.

## 3. Discussion

Amiodarone toxicity shows a variety of morphologic patterns in different organs. These morphologic patterns seem initially nonspecific and difficult to recognize, particularly when the pathologist is not alerted about amiodarone treatment. In addition, many pathologists are often unaware of the morphology of amiodarone toxicity in various organs and this is partially attributable to much of the literature being focused on investigation of biochemical, cellular and immunologic alterations induced by amiodarone rather than morphology. 

With amiodarone toxicity of the liver, Ramachandran et al. described several different morphological patterns of injury. The most frequent pattern was a steatohepatitis pattern of injury comprising of steatosis, sinusoidal fibrosis, ballooned hepatocytes with prominent hyaline, and neutrophilic satellitosis. Lysosomal inclusion bodies representing phospholipidosis were occasionally present on electron microscopy; however, the absence of phospholipidosis and steatosis has never been described. Hepatocellular necrosis and fibrosis were occasionally observed and noted to have poor prognosis [6, 12]. A granulomatous pattern of liver injury was described by Rigas et al. [13]. 

In our patient, the ballooned hepatocytes and the Mallory-Denk bodies along with the fibrosis pattern were certainly suggestive of a steatohepatitis pattern of injury, but the absence of phospholipidosis and steatosis was the most curious finding. As confirmation for the morphologic absence of phospholipidosis, electron microscopy ultrastructurally showed no lamellar lysosomal inclusion bodies. Because of the patient's history of cardiac anomalies; the extensive remodeling, damage to zone 3, and perisinusoidal fibrosis could have been attributed to the venous outflow damage from cardiac anatomic and physiologic outflow problems. The presence of the subtle ductular proliferation, hepatocyte ballooning, and Mallory-Denk formation, however, supported the steatohepatitis pattern of amiodarone toxicity more.

In describing the morphology of amiodarone toxicity in other nonhepatic organs, only a few studies which go back several decades are available. Amiodarone lung disease (ALD) is manifested as various forms of interstitial pneumonitis. Kennedy et al. showed a wide variation temporally from acute/subacute organizing lung injury to a chronic interstitial pneumonitis with fibrosis and/or organizing pneumonia. The most common pattern was a cellular interstitial pneumonitis with interstitial mononuclear inflammation, type II pneumocyte hyperplasia, alveolar lining cells with vacuolated cytoplasm, and intra-alveolar foamy macrophages. Like amiodarone toxicity in the liver, lamellar inclusion bodies were occasionally present on electron microscopy [14]. Ruangchira-Urai et al. described a more nodular form of ALD with all cases presenting with irregular masses on computed tomography (CT) scan. Microscopic sections of the macroscopic nodules showed vacuolated histiocytes massed within alveoli and basophilic necrosis amid solid inflammation. Interestingly, the presence of neutrophilic aggregates within the necrosis and also within bronchioles produced a pattern of necrotizing bronchiolitis or small abscesses that mimicked infection or Wegener's granulomatosis. Palisaded epithelioid histiocytes and multinucleated giant cells were not present [15].

In a case of progressive renal insufficiency due to amiodarone, Pintavorn and Cook described features in a renal biopsy similar to that of Fabry's disease. Such features included mildly increased mesangial matrix and mesangial hypercellularity. Dilated glomerular capillary lumina contained vacuolated histiocytes, and small inclusions were present in the visceral epithelial and mesangial cells. Electron microscopy confirmed much of the morphologic findings with thickened capillary basement membranes, widening of the lamina densa, and focal expansion of the subendothelial space. Like the other organ sites, lysosomal lamellar inclusions were present, primarily in visceral epithelial cells, but also in mesangial and tubular epithelial cells [16].

In a nerve biopsy of a one patient who developed polyradiculoneuropathy, Ruangchira-Urai et al. described focal aggregates of large histiocytes with vacuolated cytoplasm in a perineurial distribution. Like in other organs, electron microscopy showed the lamellar lysosomal inclusion bodies within these swollen histiocytes [15]. Mukhopadhyay et al. reported granulomas in the bone marrow in two cases solely attributable to amiodarone toxicity [17]. In the thyroid, Guyetant et al. reported moderate T-lymphocytic infiltration, regeneration, colloid transformation of the parenchyma, and areas of follicular disruption with numerous foamy histiocytes in the colloid. Lysosomal lamellar inclusion bodies were absent on electron microscopy in that series [18].

In conclusion, Amiodarone is a frequently used antiarrhythmic that shows various effects in different organs. The toxicity is difficult to recognize clinically due to its potential to mimic various conditions, but a biopsy is sometimes able to suggest the diagnosis. On morphology, regardless of the organ site, there appears to be a consistent presence of vacuolated cells and occasionally foamy macrophages and granulomas. Ultrastructural studies, in some cases, may be of help in showing lysosomal lamellar bodies, but the absence of these bodies does not exclude the diagnosis. It is quite plausible that limited sampling of tissue may lead to a false absence of lysosomal lamellar bodies. However, we tried to mitigate this by focusing attention during electron microscopy on the ballooned cells with heavy vacuolization where yield is perceived as the highest. Moreover, our case of amiodarone toxicity in the liver became further challenging because of the complex cardiac history, elevated ANA, and understated history of amiodarone intake. Morphologically, the absence of some characteristic features such as phospholipidosis or steatosis also added to the difficulty of arrival to the diagnosis. To our knowledge, this is the first reported case of absent phospholipidosis and steatosis in amiodarone liver toxicity. After rereview of the medication history, suggestion of amiodarone toxicity was reported. After definitive A-V node ablation with pacemaker placement, amiodarone was discontinued which resulted in stabilization of the patient's liver enzymes to normal levels.
